# Four new species of the leafhopper genus *Kapsa* Dworakowska from China (Hemiptera, Cicadellidae, Typhlocybinae), with a key to Chinese species

**DOI:** 10.3897/zookeys.212.3000

**Published:** 2012-07-30

**Authors:** Yuehua Song, Zizhong Li

**Affiliations:** 1 Institute of Entomology, Guizhou University, Guiyang, Guizhou 550025, China; 2 Institute of South China Karst, Guizhou Normal University, Guiyang, Guizhou 550001, China; 3 The State Key Laboratory Incubation Base for Karst Mountain Ecology Environment of Guizhou Province, Guiyang, Guizhou 550001, China

**Keywords:** Morphology, taxonomy, Erythroneurini

## Abstract

In the present paper, four new species, *Kapsa acuminata*, *Kapsa quadrispina*, *Kapsa puerensis* and *Kapsa yanheensis* **spp. n.** from southwest China are described and illustrated, and a key to the species recorded from China is provided.

## Introduction

The leafhopper genus *Kapsa* Dworakowska (1972) belongs to the tribe Erythroneurini (Typhlocybinae) with *Typhlocyba furcifrons* Jacobi, 1941 as its type species. Recent taxonomic work on the genus includes Chiang and Knight (1990), Dworakowska (1972, 1979, 1980, 1981a, 1981b, 1994), Dworakowska et al. (1978), Dworakowska and Sohi (1978), Sohi and Mann (1992) and Song and Li (2008). So far, twenty-four species are known in the world of which seven species of *Kapsa* have been recorded from China in the above studies. In this paper, four new species from southwest China are described and illustrated and a key to males of Chinese *Kapsa* is given. All specimens examined are deposited in the collection of the Institute of Entomology, Guizhou University, Guiyang, China (GUGC) and British Museum Natural History (BMNH).

## Taxonomy

### Kapsa

Dworakowska

http://species-id.net/wiki/Kapsa

Kapsa Dworakowska, 1972: 402; 1978: 243; 1979: 22; 1980: 186; Chiang and Knight 1990: 215; Song and Li 2008: 389.

#### Type species.

*Typhlocyba furcifrons* Jacobi, 1941

#### Description.

Dorsum beige, yellow or white. Vertex unicolorous or with pair of preapical spots or with large median apical spot. Scutellum pale with or without dark lateral triangles or entirely dark.

Head narrower than pronotum, fore margin weakly produced, broadly rounded. Forewing with outer apical cell short; hind wing submarginal vein not extended to wing apex.

Male pygofer lobe with oblique dorsolateral internal ridge, usually with sparse long fine setae on lateral surface; dorsal appendage movably articulated; ventral appendages absent. Subgenital plates with 2-6 basal macrosetae. Style apex with extension; preapical lobe prominent. Connective Y-shaped, with central lobe well developed. Aedeagus with or without processes, gonopore apical on ventral surface. Anal tube usual with basal processes.

The genus is similar *Tautoneura* Anufriev, 1969 externally (body slim, dorsum yellow or white, head and face narrow) but the forewing lacks the red dots found in *Tautoneura*. It also differs in having the male pygofer lobe with basolateral setae not distinctly enlarged, and the pygofer without ventral appendages. The genus is also similar to *Empoascanara* Distant, 1918 in the male genitalia although differing in having the subgenital plate microsetae on the dorsal margin not in groups and the style with a 2nd extension. It differs externally from *Emopoascanara* in its narrower head.

#### Distribution.

India; Nepal; Sri Lanka; China (Taiwan, Sichuan, Guizhou, Yunnan); Vietnam; Indonesia; New Guinea.

#### Key to Chinese species of the genus *Kapsa* (males)

**Table:** 

1	Aedeagus with processes (Figs 9, 15, 23, 31)	2
–	Aedeagus without processes	6
2	Aedeagus with both basal and apical processes (Figs 15, 16)	*Kapsa quadrispina*sp. n.
–	Aedeagus either with basal processes or apical processes (Figs 9, 23, 31)	3
3	Pygofer with dorsal appendage bifurcate (Figs 5, 27)	4
–	Pygofer with dorsal appendage not bifurcate (Figs 13, 20)	5
4	Aedeagus with pereatrium and basal processes moderately long (Fig. 8)	*Kapsa acuminata* sp. n.
–	Aedeagus with preatrium and basal processes long (Fig. 30)	*Kapsa yanheensis* sp. n.
5	Aedeagus with processes placed apically on shaft, bifurcate near base (Figs 22, 23)	*Kapsa puerensis* sp. n.
–	Aedeagus with processes placed medially on shaft, not bifurcate	*Kapsa biprocessa* Song & Li
6	Aedeagus with dorsal apodeme short and small, not expanded in lateral view	*Kapsa fangxianga* Song & Li
–	Aedeagus with dorsal apodeme large, greatly expanded in lateral view	7
7	Pygofer dorsal appendage short, distinctly expanded at base	*Kapsa diasonica* Chiang & Knight
–	Pygofer dorsal appendage long, not distinctly expanded at base	8
8	Gonopore long (as in Fig. 9)	9
–	Gonopore short (as in Figs 22, 23, 30, 31)	10
9	Aedeagus with preatrium short	*Kapsa arca* Song & Li
–	Aedeagus with preatrium extremely long	*Kapsa elscinta* Chiang & Knight
10	Aedeagal shaft slender and sinuate	*Kapsa dolka* Dworakowska
–	Aedeagal shaft broad and straight	*Kapsa suaoensis* Chiang & Knight

### Kapsa acuminata sp. n.

urn:lsid:zoobank.org:act:C3213F38-E901-470D-BF1C-0A6D218E5B5F

http://species-id.net/wiki/Kapsa_acuminata

Figures 1–10

#### Description.

**Figures 1–10.**
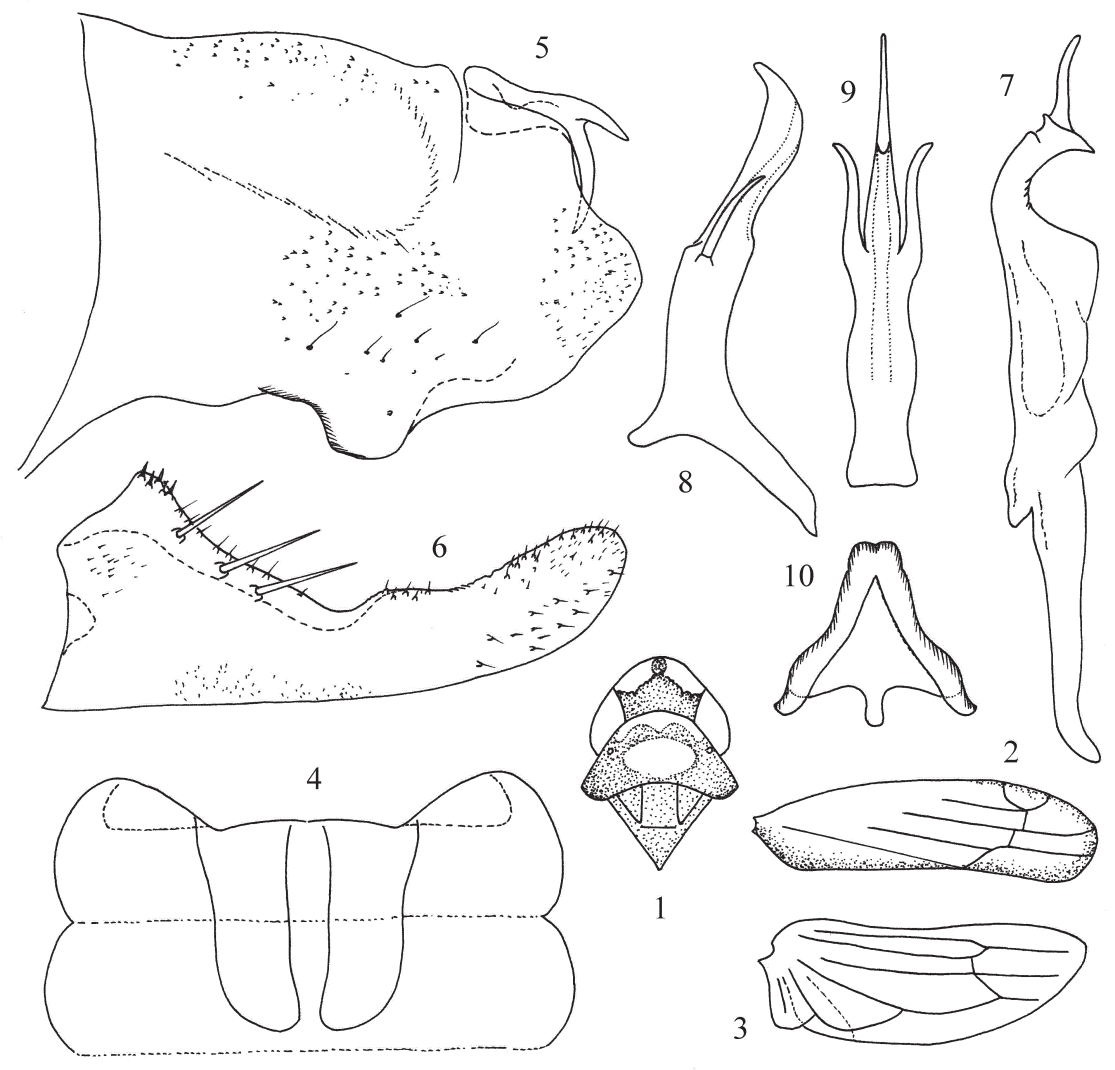
*Kapsa acuminata* sp. n. **1** Head and thorax, dorsal view **2** Forewing **3** Hind wing **4** Abdominal apodemes **5** Pygofer lobe, lateral view **6** Subgenital plate **7** Style **8** Aedeagus, lateral view **9**  Aedeagus, ventral view **10** Connective

Dorsum beige. Vertex with pair of milky yellow preapical spots; pronotum with anterior margin and median area ivory-white (Fig. 1). Forewing brownish yellow along inner and outer margin (Fig. 2).

Abdominal apodemes nearly reaching posterior margin of 4th sternite (Fig. 4).

Male pygofer with dorsal appendage bifurcate far from base (Fig. 5). Anal tube processes indistinct. Subgenital plate long, extended beyond pygofer apex, with three long macrosetae in oblique row (Fig. 6). Style elongate, preapical lobe distinct (Fig. 7). Connective Y-shaped with central lobe broad and arms short (Fig. 10). Aedeagal shaft laterally compressed distally, tapered to acute apex in ventral view (Fig. 9), with pair of processes at mid-length; gonopore long; preatrium long and dorsal apodeme short (Figs 8, 9).

#### Measurement.

Body length males 2.3~2.5 mm.

#### Type material.

*Holotype*, male, China: Guizhou Province, Mayanghe National Nature Reserve, 4 Oct. 2007, coll. Yue-hua Song. *Paratypes*: five males, same date as holotype.

#### Remarks.

The new species is similar to *Kapsa biprocessa* Song & Li (2008), but can be distinguished mainly by the aedeagus with pair of basal processes close to shaft; the more acute apex of shaft in ventral view with the gonopore longer (Figs 8, 9).

#### Etymology.

The specific epithet is derived from the Latin word “*acuminata*” which refers to the acuminate apex of aedeagus in ventral view.

### Kapsa quadrispina sp. n.

urn:lsid:zoobank.org:act:2C2E709E-3CC3-4DED-A4E4-CD274D604A76

http://species-id.net/wiki/Kapsa_quadrispina

Figures 11–17

#### Description.

**Figures 11–17.**
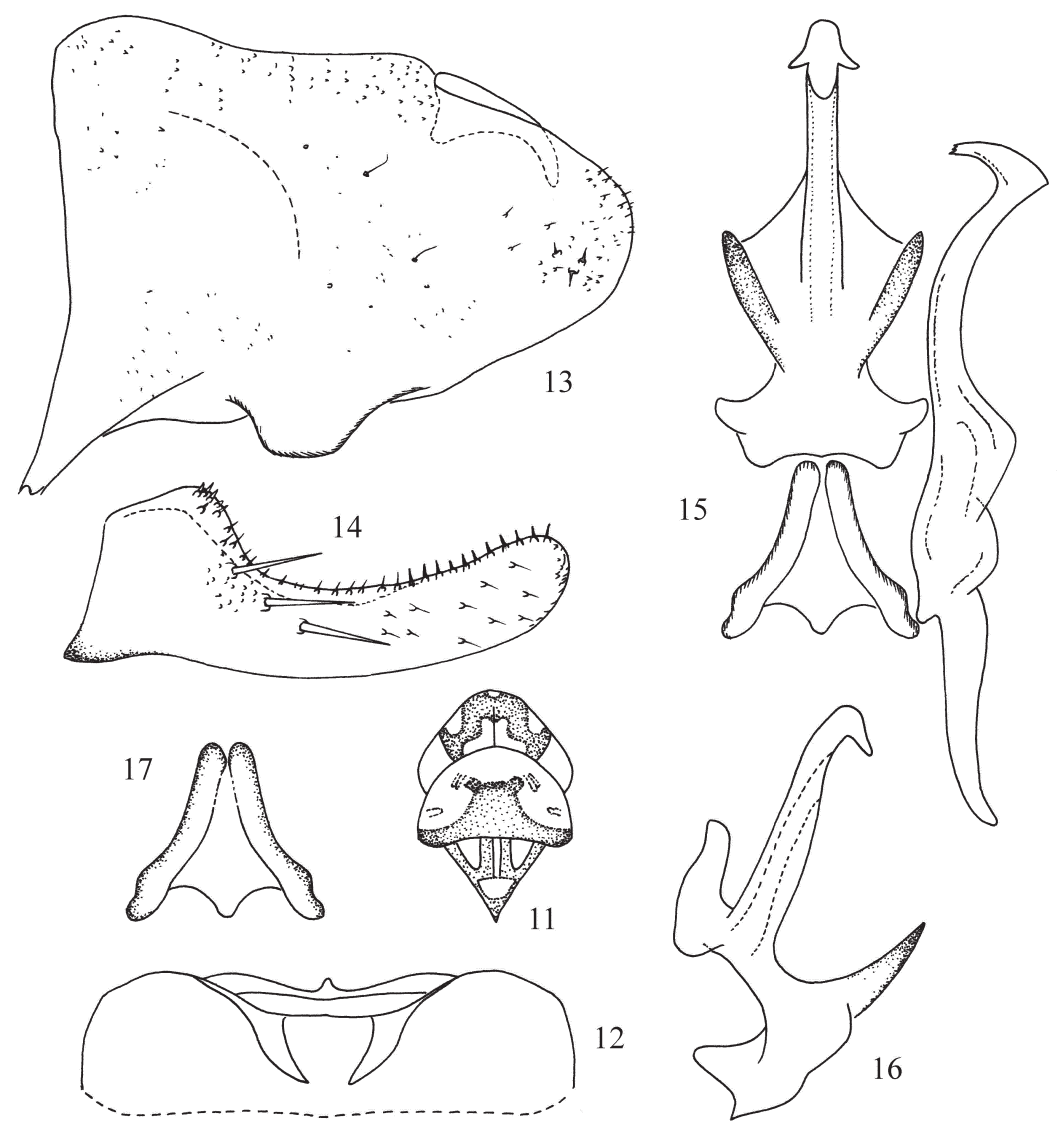
*Kapsa quadrispina* sp. n. **11** Head and thorax, dorsal view **12** Abdominal apodemes **13** Pygofer lobe, lateral view **14** Subgenital plate **15** Style, aedeagus and connective, ventral view **16** Aedeagus, lateral view **17** Connective.

Dorsum beige. Vertex with large median apical spot, brownish yellow; pronotum with median area and posterior margin, brownish yellow; scutellum with basal triangles and T-shaped streak medially, milky yellow (Fig. 11). Forewing with brochosome field orange yellow.

Abdominal apodemes slim, not exceeding 3rd sternite (Fig. 12).

Male pygofer lobe with dorsal appendage slightly curved downward in lateral view (Fig. 13). Anal tube with processes very short, indistinct. Subgenital plate with three long macrosetae in oblique row and row of short stout setae along upper margin (Fig. 14). Style elongate, with apex slightly expanded; preapical lobe prominent (Fig. 15). Connective Y-shaped with central lobe broad and arms short (Fig. 17). Aedeagus with pair of basal atrial processes, well separated from shaft, the latter with pair of short apical processes; gonopore moderately long; preatrium broad and dorsal apodeme short (Figs 15, 16).

#### Measurement.

Body length male 2.8 mm.

#### Type material.

*Holotype*, male, China: Guizhou Province, Mayanghe National Nature Reserve, at light, 30 Sep. 2007, coll. Yue-hua Song. *Paratypes*: two females, same date as holotype.

#### Remarks.

The new species is similar to *Kapsa distalis* Sohi & Mann (1992), but the aedeagus has a pair of atrial processes and apical processes (Figs 15, 16) and the dorsal pygofer appendage is not apically bifurcate (Fig. 13).

#### Etymology.

The specific name is derived from the Latin prefix “*quadri-*” and the Latin word “*spina*”, referring to the aedeagus with four processes (Figs. 15, 16).

### Kapsa puerensis sp. n.

urn:lsid:zoobank.org:act:EBB34F64-A357-4FB2-94A9-206E38FBC92E

http://species-id.net/wiki/Kapsa_puerensis

Figures 18–24

#### Description.

**Figures 18–24.**
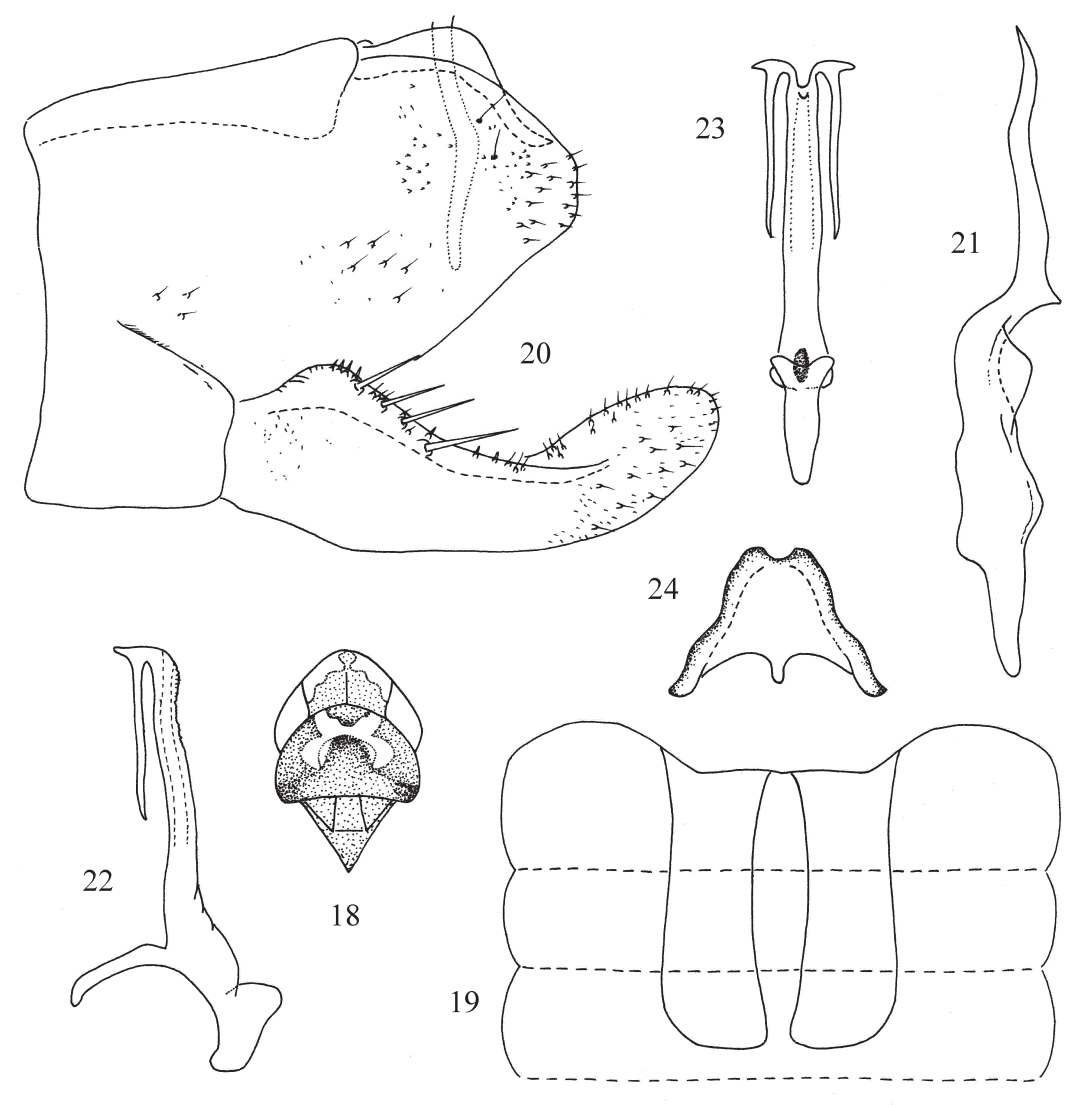
*Kapsa puerensis* sp. n. **18** Head and thorax, dorsal view **19** Abdominal apodemes **20** Male pygofer and anal tube process, lateral view **21** Style. **22** Aedeagus, lateral view **23** Aedeagus, ventral view **24** Connective.

Dorsum brownish yellow. Pair of preapical patches on vertex and medial area of pronotum, milky yellow (Fig. 18). Forewing beige.

Abdominal apodemes large, broad, extended to 5th sternite (Fig. 19).

Male pygofer with dorsal appendage expanded medially and tapering towards apex (Fig. 20). Anal tube with basal processes long, slightly curved (Fig. 20). Subgenital plate with four long macrosetae in oblique row and row of short rigid setae along upper margin (Fig. 20). Style not long, apex extremely elongate, little less than half length of style; preapical lobe distinct (Fig. 21). Connective Y-shaped with central lobe broad and arms short (Fig. 24). Aedeagal shaft with pair of apical processes, bifurcate near base, upper branch short, tooth-like; lower branch very long; gonopore short (Figs 22, 23); dorsal apodeme slender in lateral view and pretrium expanded laterally at base (Figs 22, 23).

#### Measurement.

Body length males 2.8~2.9 mm, females 2.9~3.0 mm.

#### Type material.

*Holotype*, male, China: Yunnan Province, Pu’er City, Meizihu Park, 23 July 2008, coll. Yue-hua Song. *Paratypes*: three males, two females, same date as holotype.

#### Remarks.

The new species is similar to *Kapsa decorata* Dworakowska (1981), but the aedeagus has branched apical processes, the lower one much longer than upper one (Figs 22, 23) and the preatrium is expanded laterally at base and the dorsal apodeme is slender (Fig. 22).

#### Etymology.

The specific name is named for its type locality.

### Kapsa yanheensis sp. n.

urn:lsid:zoobank.org:act:B54E04DD-B5B8-4D6F-B11A-6A09489C75A9

http://species-id.net/wiki/Kapsa_yanheensis

Figures 25–33

#### Description.

Dorsum beige. Vertex with large dark median apical spot; anterior margin of vertex and pronotum milky yellow.

Abdominal apodemes small, not exceeding 3rd sternite (Fig. 26).

Male pygofer lobe with dorsal appendage bifurcate near base, curved ventrally (Fig. 27). Anal tube processes indistinct. Subgenital plate with three long macrosetae in oblique row on lateral surface (Fig. 28). Style apex elongate, little sinuate; preapical lobe prominent (Fig. 29). Connective Y-shaped with central lobe broad and arms short (Fig. 32). Aedeagal shaft with pair of long basal processes, extending to near apex of shaft; gonopore short (Figs 30, 31); dorsal apodeme short and preatrium long (Fig. 30).

#### Measurement.

Body length males 2.5~2.7 mm, females 2.6~2.8 mm.

#### Type material.

*Holotype*, male, China: Guizhou Province, Yanhe County, Mayanghe National Nature Reserve, 30 Sep. 2007, coll. Yue-hua Song. *Paratypes*: four males, ten females, same date as holotype.

#### Remarks.

The new species is similar to *Kapsa mingorensis* (Ahmed, 1970) (see also Dworakowska et al. 1978), but the aedeagus has a pair of basal processes, without apical vestiture (Figs 30, 31) and the preatrium is not expanded in lateral view (Fig. 30).

#### Etymology. 

The new species is named for its type locality: Yanhe.

**Figures 25–33.**
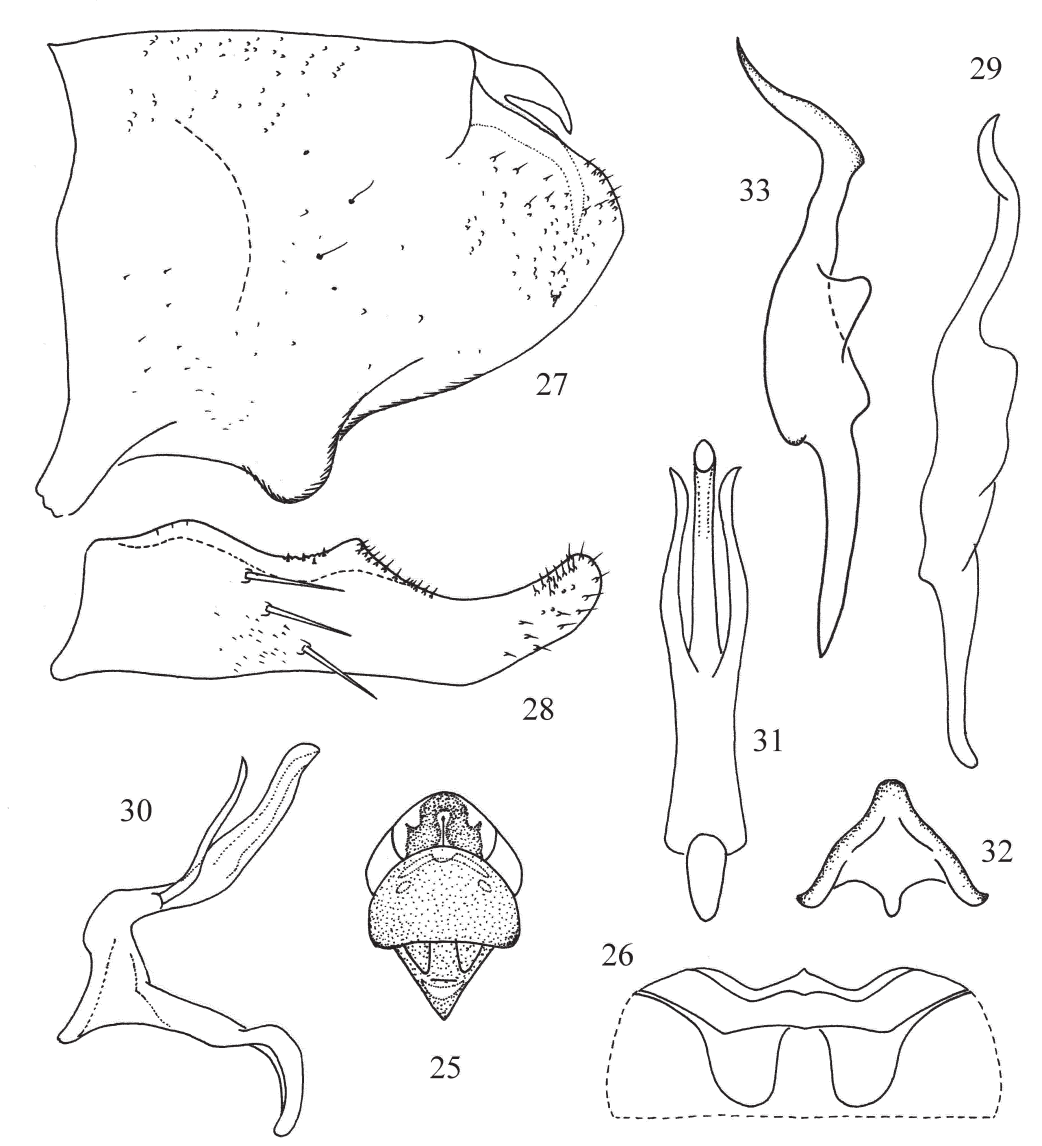
*Kapsa yanheensis* sp. n**. 25** Head and thorax, dorsal view **26** Abdominal apodemes **27** Male pygofer lobe, lateral view. **28** Subgenital plate **29** Style, lateral view **30** Aedeagus, lateral view **31** Aedeagus, ventral view **32** Connective, ventral view **33** Style, dorsal view.

## Supplementary Material

XML Treatment for Kapsa

XML Treatment for Kapsa acuminata

XML Treatment for Kapsa quadrispina

XML Treatment for Kapsa puerensis

XML Treatment for Kapsa yanheensis
